# Enhanced production of hydroxy fatty acids in *Arabidopsis* seed through modification of multiple gene expression

**DOI:** 10.1186/s13068-022-02167-1

**Published:** 2022-06-18

**Authors:** Mid-Eum Park, Kyeong-Ryeol Lee, Grace Q. Chen, Hyun Uk Kim

**Affiliations:** 1 Department of Molecular Biology, Sejong University, Seoul, Republic of Korea; 2 Department of Agricultural Biotechnology, Rural Development Administration, National Institute of Agricultural Sciences, Jeonju, Republic of Korea; 3 Western Regional Research Center, Agricultural Research Service, U.S. Department of Agriculture, Albany, CA USA; 4 Department of Bioindustry and Bioresource Engineering, Plant Engineering Research Institute, Sejong University, Seoul, 05006 Republic of Korea

**Keywords:** Diacylglycerol acyltransferase 2, Fatty acid elongase 1, Lyso-PC acyltransferase, Oleate ∆ 12-hydroxylase, Phospholipid: DAG acyltransferase 1–2, Phosphatidylcholine: DAG cholinephosphotransferase

## Abstract

**Background:**

Castor (*Ricinus communis L*.) seeds contain unusual fatty acid, hydroxy fatty acid (HFA) used as a chemical feedstock for numerous industrial products. Castor cultivation is limited by the potent toxin ricin in its seeds and other poor agronomic traits, so it is advantageous to develop a suitable HFA-producing crop. Significant research efforts have been made to produce HFA in model *Arabidopsis*, but the level of HFA produced in transgenic *Arabidopsis* is much less than the level found in castor seeds which produce 90% HFA in seed oil.

**Results:**

We designed a transformation construct that allowed co-expression of five essential castor genes (named *pCam5*) involved in HFA biosynthesis, including an *oleate* $\Delta$ *12-hydroxylase* (*FAH12*), *diacylglycerol* (*DAG*) *acyltransferase 2* (*DGAT2*), *phospholipid: DAG acyltransferase 1–2* (*PDAT1-2*), *phosphatidylcholine* (*PC*)*: DAG cholinephosphotransferase* (*PDCT*) and *Lyso-PC acyltransferase* (*LPCAT*). Transgenic *Arabidopsis* *pCam5* lines produced HFA counting for 25% in seed oil. By knocking out *Arabidopsis* *Fatty acid elongase 1* (*AtFAE1*) in *pCam5* using CRISPR/Cas9 technology, the resulted *pCam5-atfae1* lines produced over 31% of HFA. Astonishingly, the *pCam5-atfae1* line increased seed size, weight, and total oil per seed exceeding wild type by 40%. Seed germination, seedling growth and seed mucilage content of *pCam5-atfae1* lines were not affected by the genetic modification.

**Conclusions:**

Our results provide not only insights for future research uncovering mechanisms of HFA synthesis in seed, but also metabolic engineering strategies for generating safe HFA-producing crops.

**Supplementary Information:**

The online version contains supplementary material available at 10.1186/s13068-022-02167-1.

## Background

Castor seed oil contains 80–90% of ricinoleic acid (12-hydroxy-octadeca-9-enoic acid, 18:1OH), a typical hydroxy fatty acid (HFA) widely used as an industrial raw material for manufacturing high-grade lubricant, paint, coating, plastic, and pharmaceutical products [1, 2]. Castor production is hampered by the presence of deadly toxin ricin and potent allergenic 2S albumins [3–5]. Other limiting factors include narrow growth adaptation to tropical regions and labor-intensive hand-harvesting due to not simultaneously maturation of seeds [6]. As such, it is desirable to develop new HFA-producing crops that are safe and suitable for agronomic practices.

**Fig. 1:**
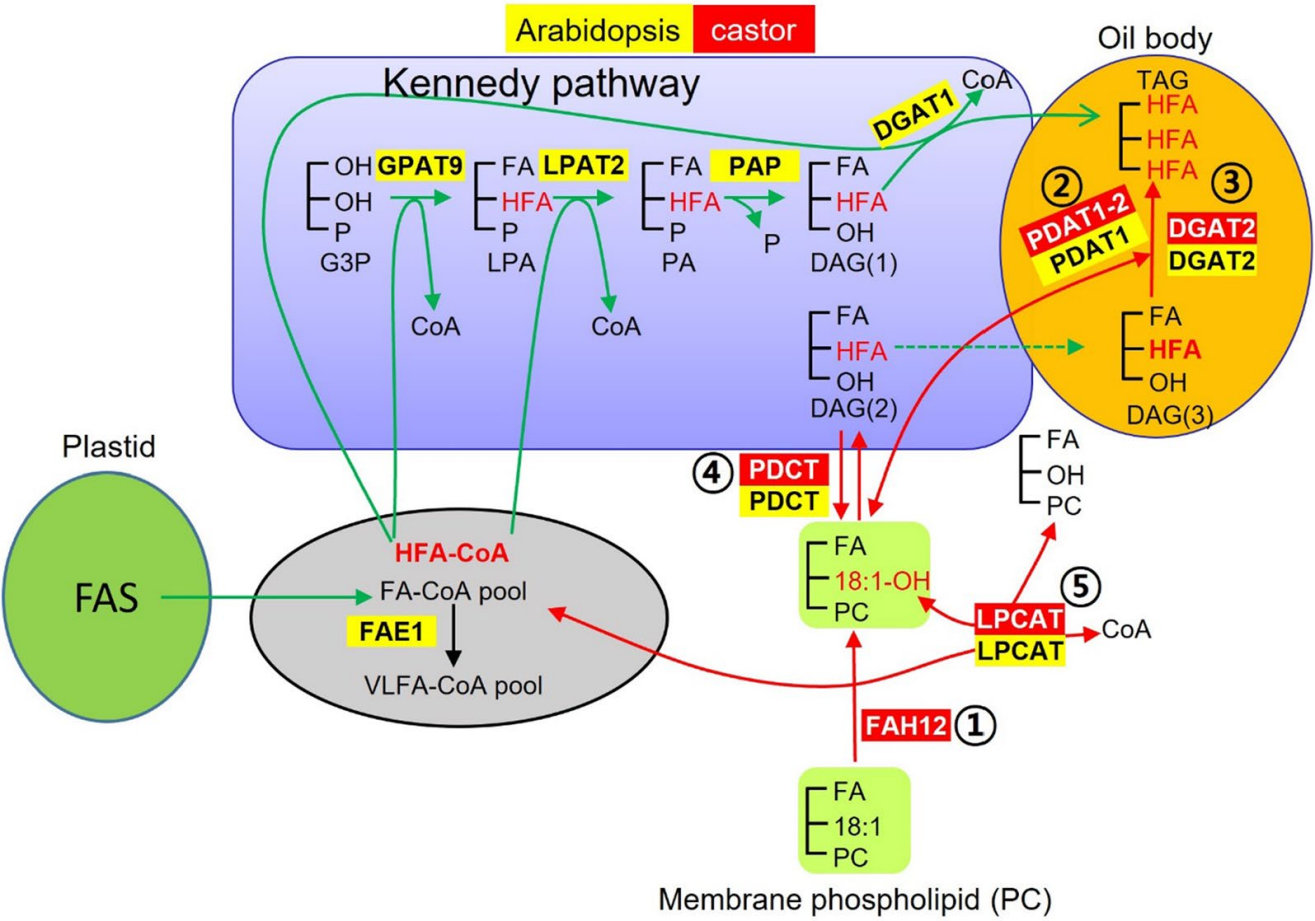
HFA-containing TAG synthesis pathways in *Arabidopsis* seeds. TAG synthesis is depicted by Kennedy pathways (green arrows) and PC-mediated pathways (red arrows). *Arabidopsis* genes are highlighted in yellow. Castor genes found to enhance HFA accumulation in *Arabidopsis* are highlighted in red. Numbers in circles are enzymatic steps targeted in this study. DAG(1) is de novo DAG. DAG(2) is initial rapid formation from PC-derived DAG [55]. DAG(3) is a bulk slowly turned over DAG pool. Dashed arrow indicates DAG(2) can be phase partitioned into DAG(3). FA numerical symbols: 18:1, oleic acid; 18:1OH, ricinoleic acid. Abbreviations: CoA, co-enzyme A; DAG, diacylglycerol; DGAT, diacylglycerol acyltransferase; FAE1, fatty acid elongase1; FAH12, oleate Δ12–hydroxylase; FAS, fatty acid synthesis; G3P, glycerol-3-phosphate; GPAT, glycerol-3-phosphate acyltransferase; LPA, lysophosphatidic acid; LPAT, lysophosphatidic acid acyltransferase; LPCAT, lysophosphatidylcholine acyltransferase; PA, phosphatidic acid; PAP, phosphatidic acid phosphatase; PC, phosphatidylcholine; PDAT, phospholipid:DAG acyltransferase; PDCT, PC:DAG cholinephosphotransferase; TAG, triacylglycerol

Pathways for seed oil (triacylglycerol, TAG) biosynthesis have been well studied. During seed development, fatty acids (FAs) are synthesized in plastids, exported to the cytosol, and activated to acyl-coenzyme As (acyl-CoAs). The acyl-CoAs are transferred into glycerol-3-phosphate (G3P) to synthesize TAG in the endoplasmic reticulum (ER) [7, 8] (Fig. 1). In the ER, TAGs are synthesized mainly through the de novo biosynthetic pathway or Kennedy pathway [7, 9–11], which consists of three sequential acylations of acyl-CoAs to a G3P backbone by glycerol-3-phosphate acyltransferase (GPAT) to produce lysophosphatidic acid (LPA), followed by LPA acyltransferase (LPAT) to generate phosphatidic acid (PA), and PA is then converted to 1,2-*sn*-diacylglycerol (DAG, or de novo DAG, DAG(1)) by PA phosphatase (PAP). Finally, the DAG is acylated by 1,2-*sn*-diacylglycerol acyltransferase (DGAT) to produce TAG (Fig. 1). In the cytosol, acyl-CoAs can also be directly incorporated into phosphatidylcholine (PC) through an acyl editing cycle [7, 8, 12–15]. Lyso-PC acyltransferase (LPCAT) is involved in the forward acylation of *sn-*2 lyso-PC using acyl-CoA and the reverse reactions of de-acylation *sn*-2 PC to yield acyl-CoA [7, 15–18]. The de-acylation of *sn*-2 PC can also occur with phospholipase A (PLA_2_)-type activity to yield a free FA, which is then activated to acyl-CoA [19]. Because the FA on the *sn*-2 PC is the substrate for FA-modifying enzymes, such as desaturases and hydroxylases, rapid de-acylation and re-acylation of PC cause the acyl-CoA pool to be enriched with modified FAs (mFAs), which can subsequently be used for TAG synthesis. Besides the Kennedy pathway, multiple routes utilizing PC lead to TAG formation. PC can be converted to DAG (PC-derived DAG, or DAG(2)) through the removal of the head group from the PC by PC:DAG cholinephosphotransferase (PDCT) [20–22]; therefore, acyl-CoAs on the PC are directed to DAG for TAG synthesis. PC-derived DAG can be produced by the reverse action of CDP-choline: DAG cholinephosphotransferase (CPT) [23], a lipase-based mechanism using phospholipase C (PLC), or phospholipase D plus PAP [24]. Because FAs in *sn*-2-PC can be modified, the conversion of PC into DAG also provides a means to increase the amount of mFAs in *sn*-2-TAG. Moreover, FA on the *sn*-2 PC can be transferred to the *sn*-3 position of DAG by phospholipid:DAG acyltransferase (PDAT) [25–27]. Recent metabolic research using *Arabidopsis* indicates that there is a third DAG pool, bulk-PC-derived DAG (or DAG(3)), a slowly turned over pool likely equilibrated by PDCT-mediated PC-DAG interconversion [28–30] (Fig. 1). AtDGAT1 produces TAG from a rapidly produced PC-derived DAG(2) pool, whereas AtPDAT1 and plant DGAT2 utilize bulk-PC-derived DAG(3) pool [28] (Fig. 1). Furthermore, there is growing evidence of existing membrane-associated complexes (or metabolons) made up of enzymes for some or all the reaction steps in a given pathway [28, 31]). The overall control of TAG biosynthesis has also been addressed for the importance of cellular, organellar, and sub-organellar localization of enzymes, structural proteins, and substrate pools [11, 32].

Unlike *Arabidopsis* and most commercial oilseeds, castor has evolved to co-ordinately synthesize and incorporate HFA (18:1OH) into the seed at 90% of TAG [33]. To explore the potential of HFA synthesis in a non-HFA producer, *Arabidopsis* has been used as a model for studying castor genes and pathways for HFA accumulation in seed [34] (Table 1). The first castor gene isolated and demonstrated to be responsible for 18:1OH synthesis is *oleate* $\Delta$ *12-hydroxylase* (*RcFAH12*) gene, which converts oleic acid (18:1) to 18:1OH at *sn*-2-PC [35]. Seed-specific expression of *RcFAH12* in *Arabidopsis* reveals four non-native HFA in TAG, 18:1OH, 18:2OH, 20:1OH and 20:2OH at 7.8%, 6.6%, 2.5% and 0.4%, respectively (total HFA at 17.3%) [36]. The results suggest that *Arabidopsis* endogenous AtFAE1 is capable of elongating 18:1OH or 18:2OH to 20:1OH or 20:2OH, respectively, or AtFAD3 is responsible for desaturating 18:1OH or 20:1OH to 18:2OH or 20:2OH, respectively [36]. To simplify the FA profile and provide more 18:1 substrate for RcFAH12, *RcFAH12* is expressed in the *Arabidopsis* *fatty acid elongase1* (*fae1*) mutant, deficient in elongation of 18:1 to 20:1 in seeds [37], and a resulted stable transgenic line is named as CL37 that accumulates 18:1OH and 18:2OH at 13.6% and 3.5%, respectively, with an average of total HFA at 17% [38]. Similarly, expression of a *FAH12* in various *Arabidopsis* mutant backgrounds results in the accumulation of total HFA at approximately 17% in seed oil [39, 40] (Table 1). Undesirably, overexpression of a *FAH12* gene in* Arabidopsis*, including CL37 decreases seed oil content and seed weight [39, 41–44] (Table 1). The limited accumulation of HFA in *Arabidopsis* and negative impact on seed total oil content and weight are explained by constrains or bottlenecks of 18:1OH flux into TAGs [20, 21, 26], β-oxidation of unutilized 18:1OH [45], and/or feedback inhibition of FA synthesis [7, 46]. In CL37, inefficient utilization of 18:1OH induces posttranslational inhibition of plastid localized acetyl-CoA carboxylase activity, resulting in a decrease of de novo FA synthesis which ultimately leads to the decreased seed oil content [7, 46]. CL37 has been used to test additional castor genes for their ability to boost HFA content in seed oil. By co-expression of various acyltransferases in CL37, total HFA levels increase to 25–34% in seed TAG [21, 26, 27, 41, 42, 47, 48] (Table 1). Overexpression of additional castor genes in CL37 including castor *DGAT2* (*RcDGAT2*) [41], *RcPDAT1A* [26] or *RcPDAT1-2* [27], and *RcPDCT* [21], not only increase HFA to 25–27% but also recover seed oil content and weight (Table 1). Overexpression of *RcLPAT2* [42, 48], as well as RcLPAT3B and RcLPATB [48], also increased HFA levels in CL37. In contrast, the expression of a castor phospholipase A2-alpha in the CL37 line decreases HFA levels [19] (Table 1). Another hypothesis of limiting HFA accumulation in transgenic *Arabidopsis* is that competition occurs between endogenous and transgenic isozymes for common FAs versus HFAs [49]. Indeed, when *RcFAH12* and *RcDGAT2* are co-expressed in a null *Arabidopsis* *dgat1-2* mutant, the HFA level is further increased to 31% in seed oil [49]. Expression of all three castor acyltransferases, *RcGPAT9, RcLPAT2* and *RcPDAT1A,* in CL37 enhances HFA up to 34% [47]. In addition, overexpression of *Arabidopsis* *WRINKLED1* (*AtWRI1*) [44], a transcription factor for fatty acid biosynthesis, or *SEIPIN1* (*AtSEI1*) [43, 50], a lipid droplet (LD) associated protein localize at the ER–LD junctions during de novo LD formation, also enhance HFA and oil contents in seeds (Table 1).

**Table 1:** Chronology of research to date for the promotion of HFA production in transgenic plant seeds

Genes	Gene source	Promoter	Host plant	HFA %	Oil content	100-seed weight	References
*FAH12*	Castor	35S	Tobacco	0.1	ND	ND	van de Loo et al. [35]
*FAH12*	Castor	Napin	*Arabidopsis*	17.3	ND	ND	Broun and Somerville [36]
*FAH12*	Castor	PfFAH12	*Arabidopsis* *fad2/fae1*	19.2	ND	ND	Smith et al. [40]
			*fad3*	18.7			
			*fad3/fae1*	7			
*FAH12*	Castor	Phaseolin	*Arabidopsis* *fae1*	17	ND	ND	Lu et al. [38]
LlinFAH12	Lesquerella, castor	PfFAH12	*Arabidopsis* WT	11.8	775 ± 34 μg/100 seeds (null segregant) 489 ± 39 μg/100 seeds^c^	ND	Dauk et al. [39]
			*fad2/fae1*	17.4	ND		
*LFAH12*			*Arabidopsis* WT	11.5	Decrease		
			*fad2/fae1*	15.6	ND		
*FAH12+DGAT2*	Castor	Phaseolin	CL37	25–27	6.30 ± 0.30μg/seed^b^; 6.70 ± 0.72μg/seed^c^	2.03 ± 0.01 mg^b^; 2.19±0.27 mg^c^	Burgal et al. [41]
*FAH12+PDAT1A*	Castor	Phaseolin	CL37	25–27	5.1μg/seed^b^; 5.44μg/seed^c^	ND	van Erp et al. [49]
*FAH12+PDAT1-2*	Castor	FAE1	CL37	25–27	207 ± 5.6μg/mgDW^b^; 228 ± 4.56μg/mgDW^c^	1.34 ± 0.061 mg^b^; 1.47 ± 0.084 mg^c^	Kim et al. [27]
*FAH12+PDCT*	Castor	Phaseolin	CL37	25–27	4.4μg/seed^b^; 5.4μg/seed^c^	ND	Hu et al. [21]
*FAH12+PLA2a*	Castor	Phaseolin	CL37	Decrease	6.79μg/seed^a^; 5.05μg/seed^b^; 5.63μg/seed^c^	ND	Bayon et al. [19]
*PDAT1A*	Castor/arabidopsis	Oleosin	*atdgat*1/CL7/RcDGAT2	31.4	No significant change compared to *dgat1*/CL7/RcDGAT2 plant	ND	van Erp et al. [49]
*FAH12+WRI1*	Castor/arabidopsis	Phaseolin	CL37	20	6.43 ± 0.18μg/seed^a^; 3.62 ± 0.04μg/seed^b^; 5.61 ± 0.05μg/seed^c^	No significant change compared to *fae1*	Adhikari and Bates [44]
*SEI1*	*Arabidopsis*	β-Conglycinin	CL37	18.3	161.2 ± 5.4μg/mg^b^; 266.6 ± 31.1 μg/mg^c^	22.1 ± 0.9 μg^a^; 15.4 ± 0.7 μg^b^; 19.1 ± 0.3 μg^c^(Single seed weight)	Lunn et al. [43]
*GPAT9+ LPAT2*	Castor	β-Conglycinin	CL37/RcPDAT1A	34	352 ± 11μg/mgDW^a^; 197 ± 9.8μg/mgDW^b^; 351 ± 20μg/mgDW^c^	ND	Lunn et al. [47]
*GPAT9+LPAT2+DGAT2*	Castor	RcGPAT9–glycinin-1, RcLPAT2–β-conglycinin, RcDGAT2-2S albumin	CL37	Increase	24.2 ± 1.2%^b^; 34.2 ± 1.8%^c^(LPAT2); 29.6 ± 2.1%^c^ (GPAT9 + LPAT2 + DGAT2) (FAME of dry weight)	ND	Shockey et al. [41]
*RcLPAT1, RcLPAT2, RcLPAT3A, RcLPAT3B, RcLPAT4, RcLPAT5, RcLPATB*	Castor	Phaseolin	CL37	RcLPAT2—17.8 RcLPAT3B—20.1 RcLPATB—19.1	ND	ND	Kim et al. [48]
*pCam5 (RcFAH12, RcDGAT2, RcPDAT1-2, RcPDCT, RcLPCAT)*	Castor	RcFAH12, RcDGAT2–Phaseolin RcPDAT1-2, RcPDCT–FAE1 RcLPCAT–Napin	*Arabidopsis* WT	26	216.48 μg/mgDW^b^; 3.68 μg/seed^b^; 227.1 μg/mgDW^c^, 3.9 μg/seed^c^; 254.72 ug/mgDW^d^; 5.5 µg/seed^d^	1.7 mg^b^;1.72 mg^c^; 2.16 mg^d^	This work
*pCam5-AtFAE1*	CRISPR	Egg-cell specific promoter	pCam5	31	214.22 μg/mgDW^c^, 6.54 μg/seed^c^;	3.06 mg^c^	

*ND* not determined, *DW* dry weight

^a^*fae1*

^b^CL37

^c^Transgenic

^d^WT (COL-0)

As described above, no more than four castor genes have been simultaneously co-expressed in *Arabidopsis*. To further enhance HFA production, we co-expressed five castor genes, *RcFAH12 RcDGAT2, RcPDAT1-2, RcPDCT,* and *RcLPCAT*, in *Arabidopsis*, which resulted in *pCam5* lines. Through CRISPR/Cas9 genome editing, we deleted *AtFAE1* in *pCam5*, generating *pCam5-atfae1* lines. We found that *pCam5-atfae1* lines increased not only HFA content in seeds but also seed size and weight dramatically. Therefore, *pCam5-atfae1* lines contain the highest amount of HFA per *Arabidopsis* seed ever reported. The mechanisms underlying the enhancement of HFA production and seed development were discussed.

## Results

### Analysis of transgenic *ArabidopsispCam5* expressing five genes from castor

To maximize HFA production in transgenic plants, we included the most critical gene, *RcFAH12*, which is directly responsible for converting 18:1 to 18:1OH in developing seeds [35]. We also included *RcDGAT2*, *RcPDAT1-2* and *RcPDCT*, because these genes have been demonstrated to facilitate the channeling of 18:1OH to TAG in transgenic *Arabidopsis* through various pathways [21, 26, 27, 41] (Fig. 1). Besides, we decided to include *RcLPCAT*, as biochemical evidence indicates that it is involved in a rapid acyl-editing between HFA–CoA and HFA–PC [7, 15–18] (Fig. 1).

**Fig. 2:**
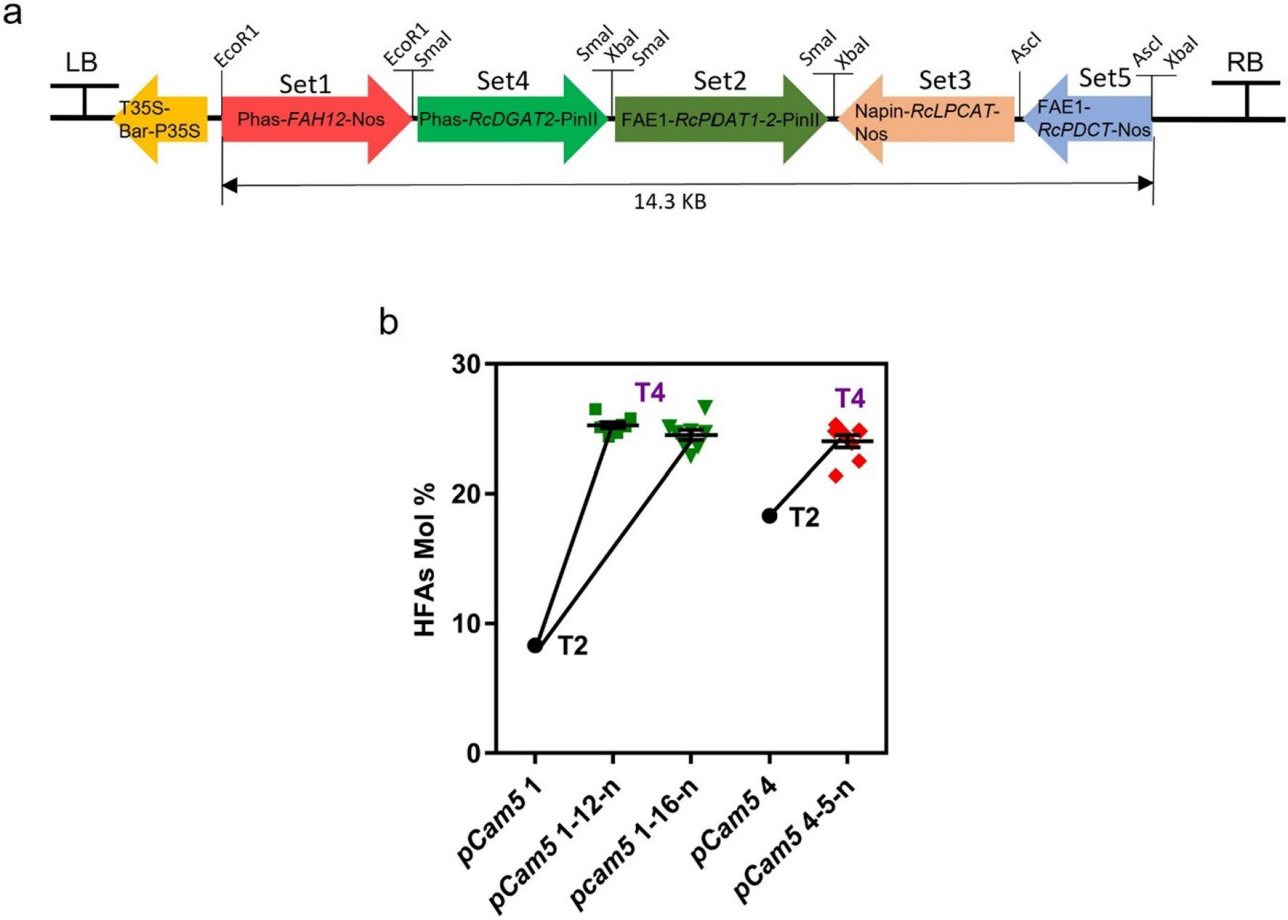
HFA content of *pCam5* vector transformants. **a** T-DNA structure of pCam5 vector of five castor gene sets (promoter–gene–terminator). Set1, *Phas-FAH12-Nos* (2986 bp); Set2, *FAE1-RcPDAT1-2-PinII* (3324 bp); Set3, *Napin-RcLPCAT-Nos* (2794 bp); Set4, *Phas-RcDGAT2-PinII* (2989 bp); Set5, *FAE1-RcPDCT-Nos* (2050 bp). Each set was sequentially cloned into pCambia3300 vector. The restriction enzyme sites used for cloning are marked on the vector. The size of entire five expression sets is 14.3 kb, and the vector backbone of pCambia 3300 is 8.4 kb, resulting in pCam5 vector in size of 22.7 kb. Phas, phaseolin promoter (1500 bp); FAE1, Fatty acid elongase 1 promoter (946 bp); Napin, napin promoter (1152 bp); Nos, Nos terminator (255 bp); PinII, PinII terminator (406 bp); FAH12 (1164 bp), DGAT2 (1023 bp), RcPDAT1-2 (1,983 bp), LPCAT (1398 bp), PDCT (838 bp), LB, Left border; RB, Right Border. **b** HFA content in T2 and T4 seeds generations of *pCam5* transformants

**Fig. 3:**
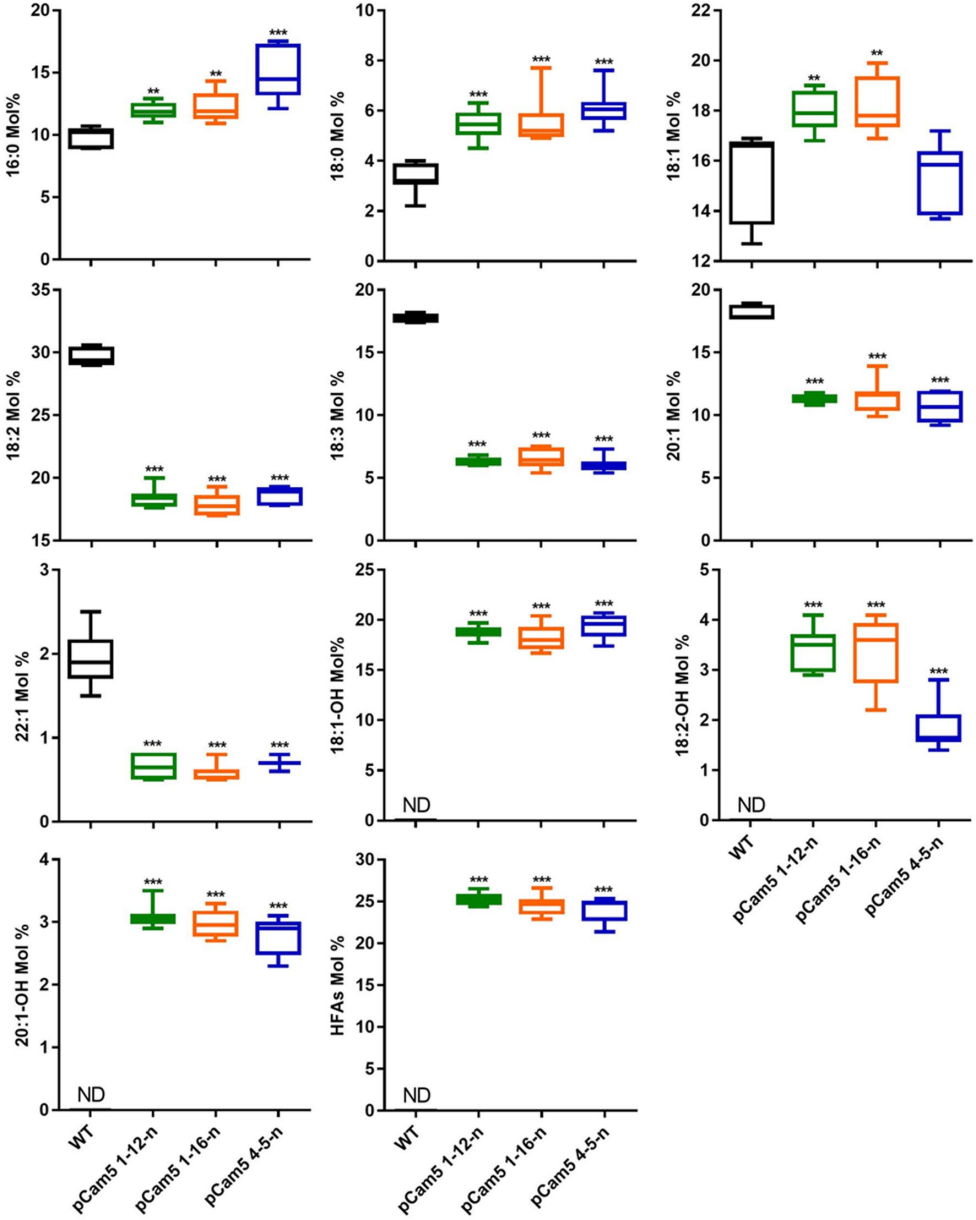
Fatty acid composition of *pCam5* transgenic T4 seeds. Comparison of changes according to fatty acid composition of wild-type seeds and *pCam5* transgenic seeds of three independent lines (*pCam5* 1–12-n, *pCam5* 1–16-n, and *pCam5* 4–5-n, Additional file 1: Table S3). Error bars represent SEM of the mean. Statistical analysis is one-way ANOVA with Dunnett’s multiple comparison test (**p < 0.01, ***p < 0.001). ND; not detected

We constructed these five genes into one transfer DNA (T-DNA) and designated it as *pCam5* (Fig. 2a). Transgenic *Arabidopsis* carrying *pCam5* T-DNA would allow the expression of these five genes simultaneously. Transgenic lines resistant to BASTA herbicide (indicating carrying *pCam5* T-DNA) were analyzed for FA composition. As HFA is our targeted metabolite, we selected the lines with the highest amount of HFA in seeds. Four T_1_ *pCam5* transformants were obtained. T_2_ seeds harvested from these four plants were analyzed for FA composition. Compared with WT which does not produce HFA, the transgenics produced three HFAs, 18:1-OH, 18:2-OH, and 20:1-OH, at 3.9–14.7%, 1.3–2.3%, and 0.4–2.2%, respectively (Additional file 1: Table S1). Line 1 (*pCam5* 1) and line 4 (*pCam5* 4) contained relatively higher total HFA at 8.3% and 18.3%, respectively (Additional file 1: Table S2). As T_2_ seeds are segregating populations, 13 T_3_ off-springs from *pCam5* 1 lines and 20 T_3_ off-springs from *pCam5* 4 lines were analyzed for FA compositions to identify homozygous individuals. As shown in Additional file 1: Table S2, total HFA contents in these T_3_ seeds ranged from 8.3% (*pCam5* 1–7) to 22.5–23% (*pCam5* 1–12, *pCam5* 1–16) among *pCam5* 1 lines, and from 12.0% (*pCam5* 4–11) to 22.8% (*pCam5* 4–5, *pCam5* 4–12) among *pCam5* 4 lines. Noticeably, the total HFA level in these lines can be roughly grouped into low levels (8.3–16.9%) or high levels (22.5–23%) (Additional file 1: Table S2). The results suggested that these five transgenes were either hemizygous or homozygous. The top T_3_ seeds from *pCam5* 1–12, *pCam5* 1–16, and *pCam5* 4–5 were grown to obtain T_4_ generation seeds, and eight T_4_ off-springs from each of these top lines were examined for their FA composition. As anticipated, total HFA contents were comparable among these off-springs, showing 24.4–26.5%, 22.9–26.6%, and 21.4–25.3%, respectively (Additional file 1: Table S3, Fig. 2b). The results indicated that *pCam5* 1–12-8, *pCam5* 1–16-7, *pCam5* 1–16-8 and *pCam5* 4–5-2 were homozygous lines. For each specific FA composition, HFAs accumulated 16.7–20.7% for 18:1OH, 1.4–4.1% for 18:2OH, and 2.3–3.5% for 20:1OH in transgenic lines, *pCam5* 1–12-n, *pCam5* 1–16-n, and *pCam5* 4–5-n, respectively (Fig. 3). Other FA levels were also changed: linoleic (18:2), α-linolenic (18:3), 11-eicosenoic (20:1), erucic (22:1) acids decreased from 29.7% to 17–20%, 17.8% to 5.4–7.5%, 18.1% to 9.2–13.9%, 1.9% to 0.5–0.8%, respectively (Fig. 3), palmitic (16:0) and stearic (18:0) acids increased from 9.8% to 10.9–17.5% and from 3.3% to 4.5–7.7%, respectively (Fig. 3). 18:1 level was comparable between WT (15.6%) and *pCam5* 4–5–n (15.4%), but the level increased in *pCam5* 1–12–n (17.9%) and *pCam5* 1–16–n (18.2%) (Fig. 3). Continued analysis on T_5_ seeds from *pCam5* 1–12-8-n, *pCam5* 1–16-7-n, *pCam5* 1–16-8-n and *pCam5* 4–5-2-n revealed no significant changes in FA composition compared with their corresponding T_4_ seeds (Additional file 1: Table S4, Fig. 2b) indicating that these five transgenes were stably inherited. Compared with the CL37 [38], which accumulated total HFA at 15.8–16.2% (Additional file 1: Table S4), a homozygote T_5_ line (*pCam5* 1–16-7–2) accumulated approximately 9% more HFA showing 25% (Additional file 1: Table S4). To verify the expression of these five transgenes, Reverse transcription PCR (RT–PCR) and quantitative RT–PCR (RT–qPCR) were performed for samples from developing seeds of *pCam5* 1–16-8 at various developmental stages. All five transgenes were expressed during seed development, where *RcFAH12* and *RcDGAT2* had a bell-shaped pattern and *RcPDAT1-2*, *RcPDCT* and *RcLPCAT* rose sharply at late stages (Additional file 2: Fig. S1a, b).

### Analysis of *pCam5-atfae1* lines produced by knock-out *ArabidopsisFAE1* in *pCam5* transgenic background through genome editing

**Fig. 4:**
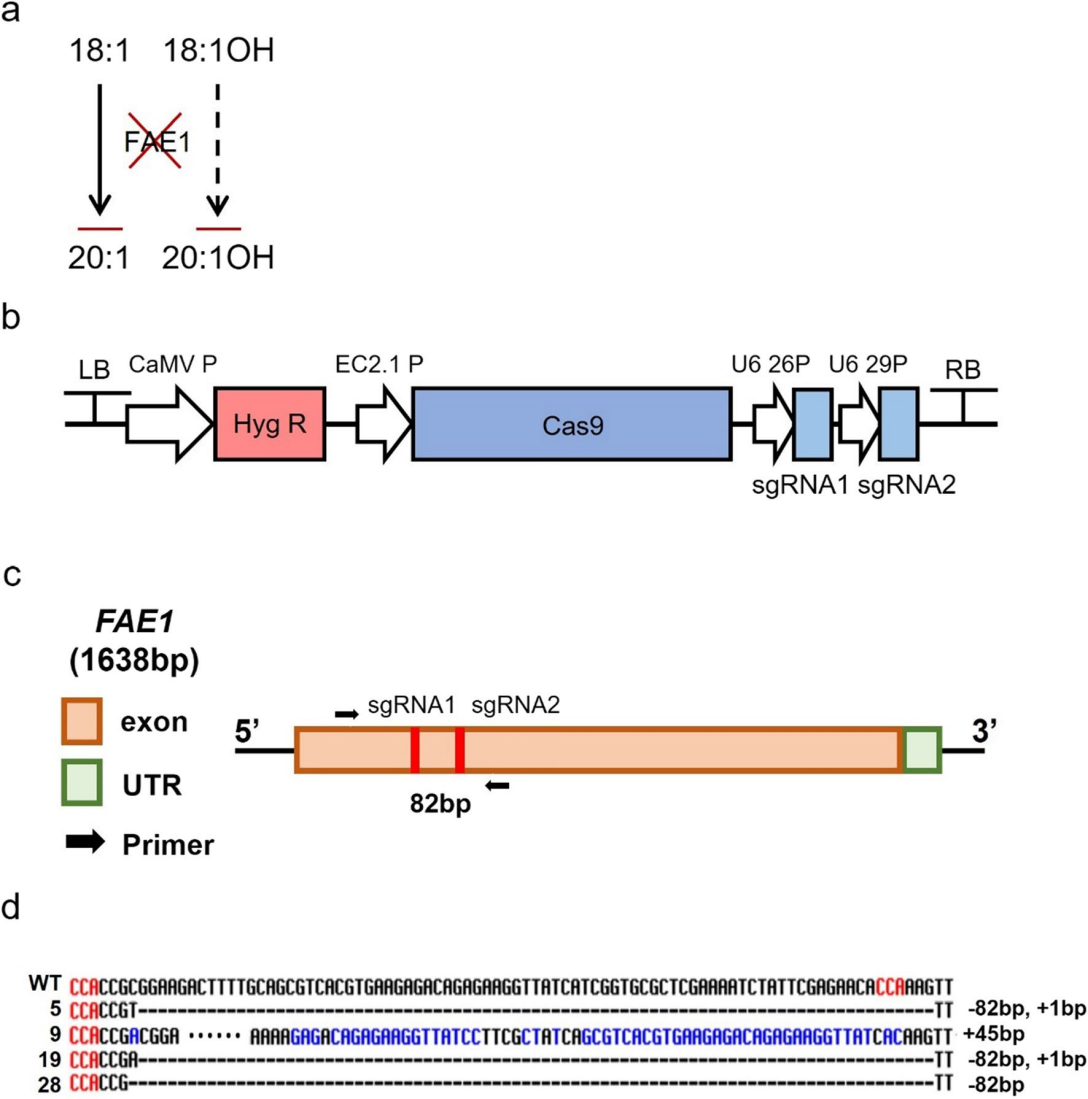
Generation of *FAE1* knockout using CRISPR/Cas9 system in *pCam5* transgenic line. **a** Fatty acid synthesis by FAE1 in transgenic *Arabidopsis* expressing RcFAH12. A solid arrow indicates strong flux, dashed arrow indicates weak flux. **b** CRISPR/Cas9 vector map for knockout of *FAE1* gene in *Arabidopsis*. HygR, hygromycin resistance gene is driven by CaMV promoter; *Cas9* gene is driven by Egg cell-specific promoter; sgRNA is driven by U6 promoter; LB/RB, left border, right border; CaMV P, Cauliflower mosaic virus gene promoter; EC2.1 P, Egg cell-specific promoter; U6 26P, U6 29P, U6 promoter. **c** Schematic diagram of design for sgRNAs in *FAE1* gene. Two single guide RNA (sgRNA1, 2) positions for *FAE1* gene editing. To identify the deletion pattern of the *FAE1* gene, two primers are designed. **d** Sanger sequencing result of four independent *pCam5-atfae1* lines with addition or deletion of *FAE1* gene. Red color indicates PAM(NGG) sequence and blue color indicates mismatch sequence compared to wild type

**Fig. 5:**
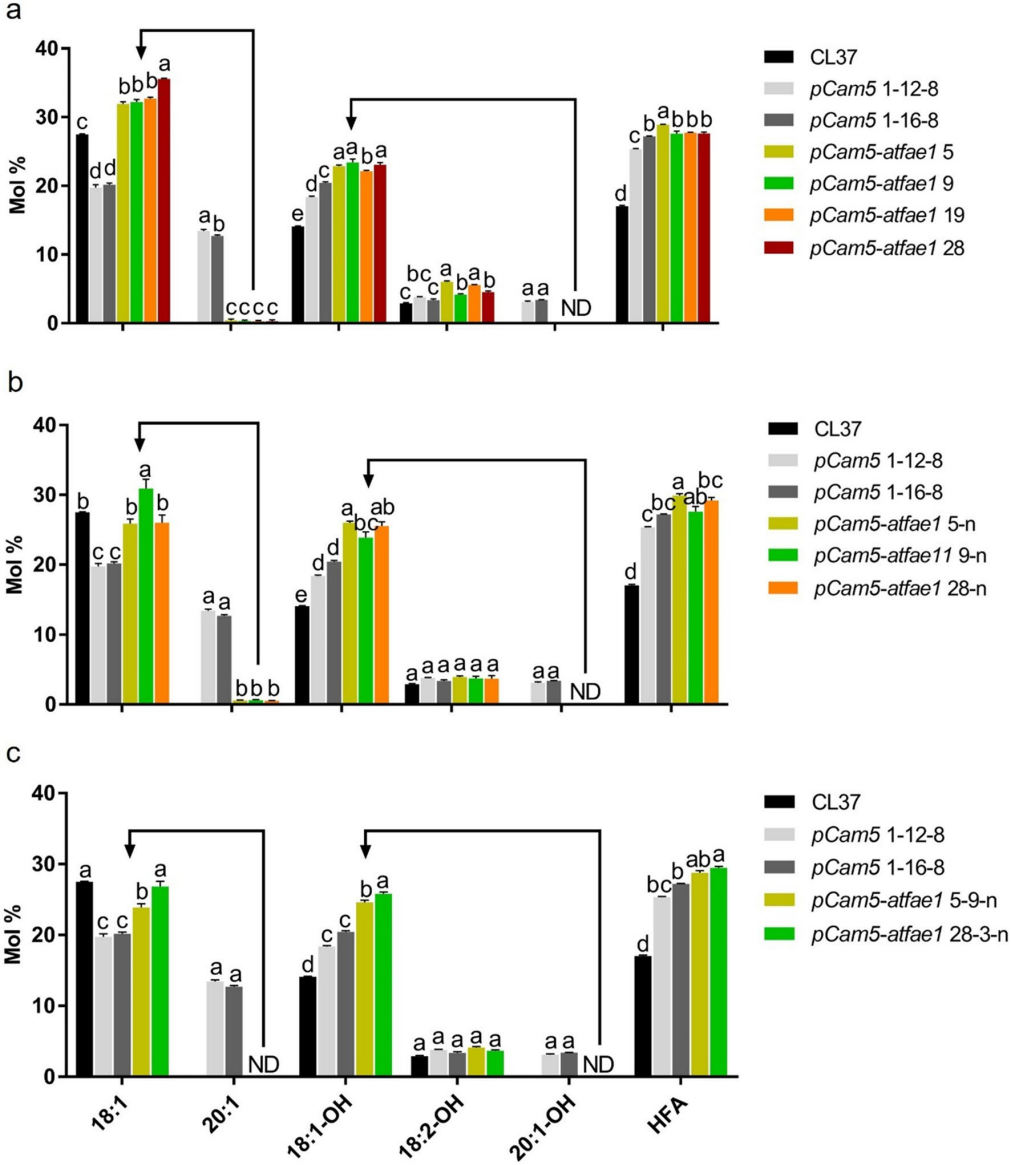
Fatty acid analysis of transgenic seeds with knock-out of *FAE1* in *pCam5* lines. *pCam5* 1–12-8 and *pCam5* 1–16-8 are controls. Fatty acid composition in seeds of *pCam5-atfae1* 5, 9, 19, and 28 at T2 (**a**), T3 (**b**), and T4 (**c**) generations. Error bars represent SEM of the mean. Statistical significance is indicated by different letters and tested by one-way ANOVA with Tukey’s multiple comparison test (*p < 0.05). ND; not detected

FAs 20:1 and 20:1OH are produced through elongation of 18:1 and 18:1OH, respectively, by *FAE1* [51, 52] (Figs. 1, 4a). Knock-out (KO) *FAE1* would block the formation of 20:1 and 20:1OH and consequently, increase 18:1 and 18:1OH accumulation. As 18:1 is the substrate for FAH12 to produce 18:1OH, the increased 18:1 could also contribute to 18:1OH accumulation (Fig. 1). To test our hypothesis, we applied CRISPR/Cas9 technology along with egg-specific Cas9 expression system [53] (Fig. 4b) and knocked out *AtFAE1* in two independent homozygous lines, *pCam5* 1–12 and *pCam5* 1–16 (Additional file 1: Table S4). Thirty-five transformants showing resistance to hygromycin were selected. PCR was performed on the leaf genomic DNA using a primer containing the predicted mutation region of the *FAE1* gene, and lines having a PCR band smaller than that of the wild-type *FAE1* gene were selected. These DNA–PCR products were subjected to Sanger sequencing (Fig. 4c). As a result, we found four independent *fae1* knock-out lines in which the *FAE1* gene was deleted at 82 bp, or added 1 bp in A or T with a deletion of 82 bp*,* or added at 45 bp, and we designated these four lines as *pCam5-atfae1* 5, *pCam5-atfae1* 9, *pCam5-atfae1* 19, and *pCam5-atfae1* 28, respectively (Fig. 4d). Compared with the background seeds (*pCam5* 1–12-8 and *pCam5* 1–16-8) which produced 18:1 and 20:1 at averages of 19.7–20.2% and 13.4–12.7%, respectively, *pCam5-atfae1* lines (T_2_ generation) increased 18:1 to 32.0–35.6% and decreased 20:1 to 0.3–0.5% (Fig. 5a, Additional file 1: Table S5). For HFAs, 18:1OH and 18:2OH increased slightly from averages of 18.4–20.4% and 3.4–3.8%, respectively, in *pCam5* lines to averages of 22.2–23.4% and 4.2–6.0%, respectively, in *pCam5-atfae1* lines (Fig. 5a, Additional file 1: Table S5); 20:1OH dropped from averages of 3.1–3.4% in background *pCam5* lines to 0% in *pCam5-atfae1* lines (Fig. 5a, Additional file 1: Table S5). We observed a small increase in total HFAs content from an average of 25.3–27.2% in *pCam5* background to averages of 27.6–28.9% in T_2_ generation of *pCam5-atfae1* lines (Fig. 5a, Additional file 1: Table S5). As described in the introduction, the CL37 line [38] is a stable transgenic *Arabidopsis* expressing *RcFAH12* in *fae1* mutant background that eliminates almost all 20:1 and 20:1OH. We compared FA compositions between CL37 and T_3_ *pCam5-atfae1* lines. As shown in Fig. 5b and Table S6, CL37 accumulates 18:1OH and 18:2OH at averages of 14.1% (± 0.16% SD) and 2.9% (± 0.12% SD), respectively, while the *pCam5-atfae1* lines accumulated 18:1OH and 18:2OH at averages of 24.1–26.2% and 3.7–3.9%, respectively (Additional file 1: Table S6). *pCam5-atfae1* 5–1 showed a highest level of HFA at 31.9% (Additional file 1: Table S6). Furthermore, the HFA content in T_4_ generation of *pCam5-atfae1* lines was examined, and the results showed that 18:1OH and 18:2OH levels were accumulated at similar levels to that of T_3_ seeds (Fig. 5c, Additional file 1: Table S7). For common FAs, CL37 contains little higher contents in 16:0, 18:2, and 18:3 at 18.0%, 23.8% and 7.1%, respectively, than that of *pCam5-atfae1* lines at averages of 12.1–12.4%, 18.5–21.2% and 5.2–5.8%, respectively (Additional file 1: Table S7). CL37 had 18:0 and 18:1 at 6.0% and 27.5%, respectively, comparable to that of *pCam5-atfae1*, showing averages at 6.3–6.4% and 23.9–26.9%, respectively (Additional file 1: Table S7). For non-HFA, the disappearance of 20:1 in *pCam5-atfae1* coincided with the increases of 16:0 from 9.6–9.7% to 12.1–12.4%, 18:1 from 19.7–20.2% to 23.9–26.9%, and 18:2 from 17.1–18.5% to 18.5–21.2% (Additional file 1: Table S7). To verify that transgenic genes were expressed in *pCam5-atfae1* 5–9 line, RT–PCR and RT–qPCR were performed for samples from developing seeds at various stages. We found that all five genes were expressed during stage one to stage six of *pCam5-atfae1* 5–9 line (Additional file 2: Fig. S1c, d).

### Comparison of oil content, seed size, and seed germination among wild type, CL37, *pCam5* 1–16-7–2, and *pCam5-atfae1* 28–3-6

**Fig. 6:**
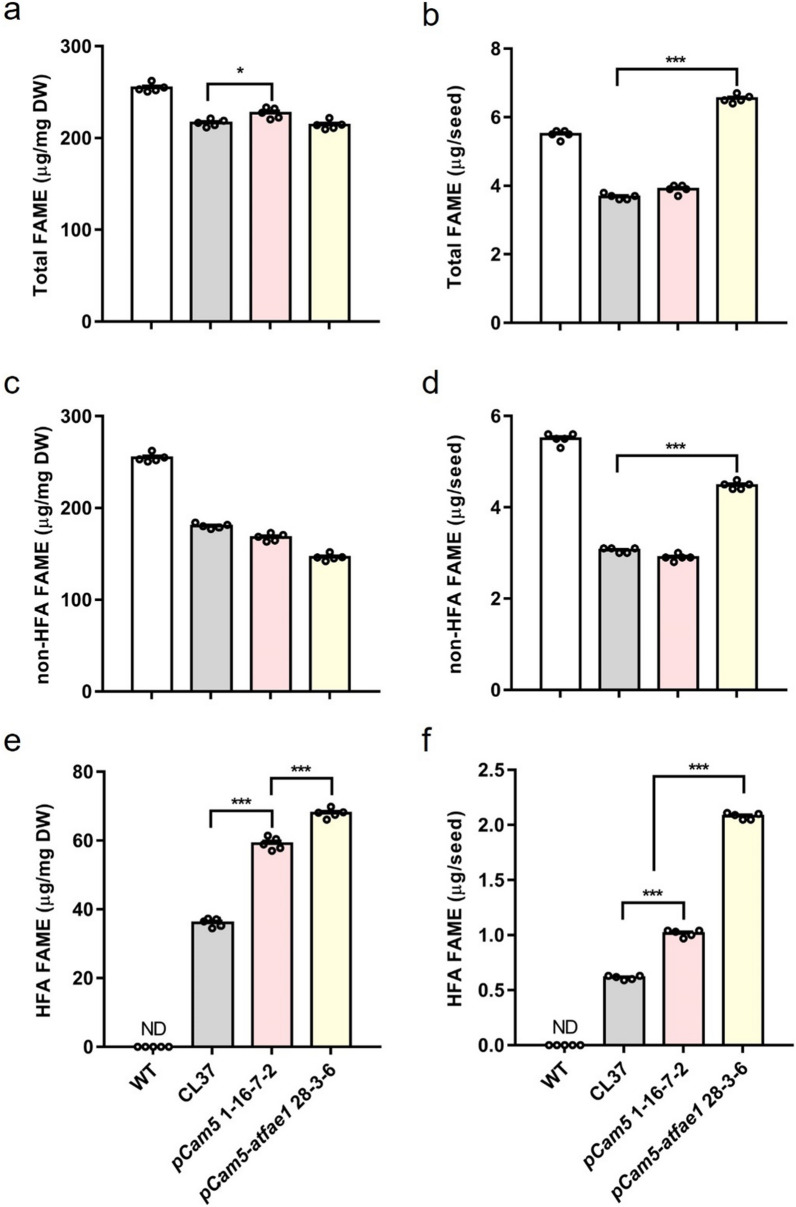
Comparison of oil (total FAs) contents among transgenic lines (*pCam5* and *pCam5-atfae1*), wild type (WT), and CL37. **a** Total FAME per mg DW. **b** Total FAME per seed. **c** non-HFA FAME per mg DW. **d** non-HFA FAME per seed. **e** HFA FAME per mg DW. **f** HFA FAME per seed. The results are measured in five technical replicates. Error bars represent SEM of the mean (*n* = 5). Statistical analysis is one-way ANOVA with Turkey’s multiple comparison test (*p < 0.05, ***p < 0.001). ND; not detected

To determine the oil content of the seeds, the total fatty acid methyl ester (FAME) content was measured by gas chromatography (GC) for wild type (WT, 0% HFA), and three transgenics, CL37 (17% HFA), *pCam5* 1–16-7–2 (25.3% HFA), and *pCam5-atfae1* 28–3-6 (30.5% HFA). WT contained a little higher level of FAME per mg dry weight (DW) (254.72 µg/mg) than that of *pCam5* 1–16-7–2 (227.1 µg/mg DW), CL37 (216.48 µg/mg DW), and *pCam5-atfae1* 28–3-6 (214.22 µg/mg DW) (Fig. 6a). However, *pCam5-atfae1* 28–3-6 had significant higher amount of total FAME content per seed (6.54 µg/seed) than that of CL37 (3.68 µg/seed), and *pCam5* 1–16-7–2 (3.9 µg/seed) (background control of *pCam5-atfae1* 28–3-6) (Fig. 6b). WT had 5.5 µg/seed of FAME (Fig. 6b). To examine the effect of genes on HFA content, we calculated the non-HFA–FAME and HFA–FAME. WT contains the most non-HFA–FAME at 254.72 µg/mg DW followed by CL37 (180.36 µg/mg DW), *pCam5* 1–16-7–2 (167.98 µg/mg DW), and *pCam5-atfae1* 28–3-6 (146.26 µg/mg DW) (Fig. 6c). In term of non-HFA–FAME content per seed, WT also contains the most at 5.5 µg/seed, however, followed by *pCam5-atfae1* 28–3-6 (4.48 µg/seed), CL37 (3.06 µg/seed), and *pCam5* 1–16-7–2 (2.9 µg/seed) (Fig. 6d). Regarding HFA–FAME, *pCam5-atfae1* 28–3-6 contained the most at 67.94 µg/mg, or 2.06 µg/seed, followed by *pCam5* 1–16-7–2 (59.1 µg/mg, 1 µg/seed) and CL37 (36.12 µg/mg, 0.6 µg/seed) (Fig. 6e, f).

**Fig. 7:**
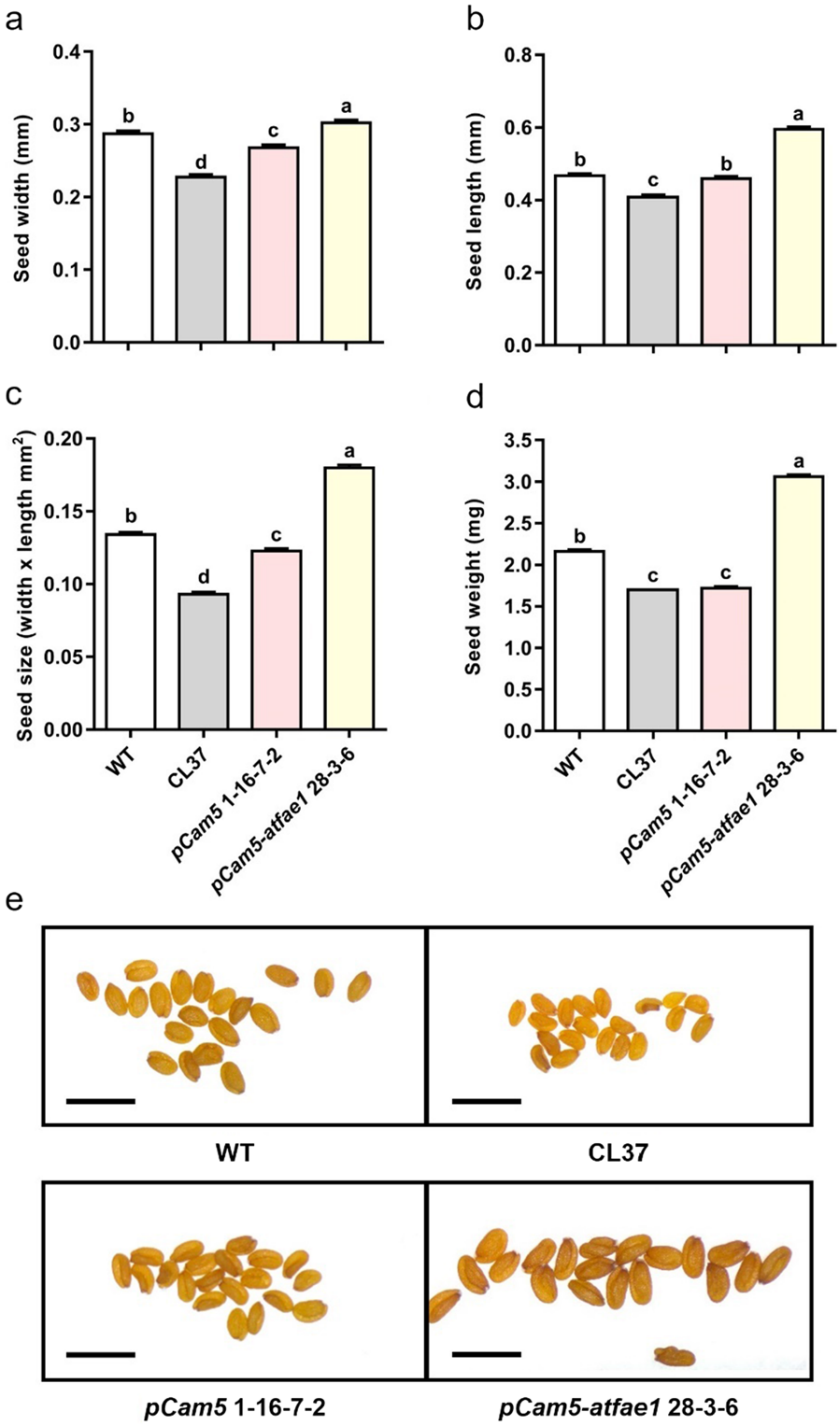
Characterization of seeds in transgenic *pCam5* and *pCam5-atfae1* lines. **a** Seed width. **b** Seed length. **c** Seed size (width × length) of wild type, CL37, *pCam5* 1–16-7–2, and *pCam5-atfae1* 28–3-6. The results are measured in triplicate (*n* = 20). **d** Seed weight was measured in five technical replicates (*n* = 100). **e** Image of *Arabidopsis* seed. Scale bar = 1 mm. Error bars represent SEM of the mean. Statistical significance is indicated by different letters and tested by one-way ANOVA with Tukey’s multiple comparison test (*p < 0.05)

The size and weight of WT, CL37, *pCam5* 1–16-7–2 and *pCam5-atfae1* 28–3-6 were measured. The average width of WT was 0.29 mm, slightly wider than CL37 (0.23 mm) and *pCam5* 1–16-7–2 (0.27 mm). However, *pCam5-atfae1* 28–3-6 increased to 0.30 mm (Fig. 7a). Seed length did not differ between WT and *pCam5* 16–7-2, but seed length of *pCam5-atfae1* 28–3-6 was 0.59 mm, significantly larger than that of CL37 (Fig. 7b). The seed size (estimated by multiplying the length and width of the seed) of *pCam5-atfae1* 28–3-6 was significantly bigger than that of WT, CL37 and *pCam5* 1–16-7–2 (Fig. 7c). Seed sizes at descend order are *pCam5-atfae1* 28–3-6 (0.18 mm^2^) > WT (0.13 mm^2^) > *pCam5* 16–7-2 (0.12 mm^2^) > CL37 (0.09 mm^2^). The 100 seed weight of *pCam5-atfae1* 28–3-6 was 3.06 mg, significantly heavier than that of WT (2.16 mg/100 seeds), pCam5 1–16-7–2 (1.72 mg/100 seeds), and CL37 (1.7 mg/100 seeds) (Fig. 7c, d, e). Compared with CL37, *pCam5-atfae1* 28–3-6 increased by 100% in seed size and weighed 80% heavier. When compared with WT, *pCam5-atfae1* 28–3-6 exceeded by 40% both in seed size and weight.

**Fig. 8:**
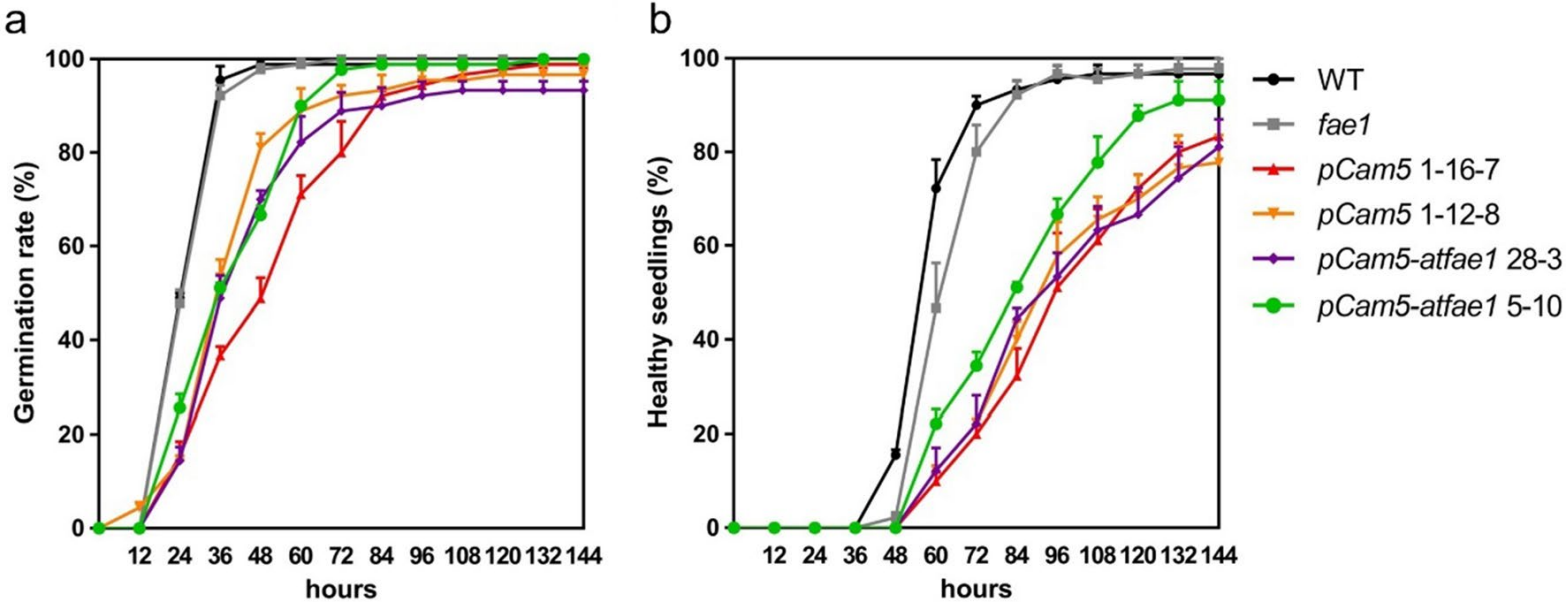
Germination rate and healthy seedlings in *pCam5* and *pCam5-atfae1* transgenic lines. **a** Germination rate. **b** Percentage of healthy seedlings with fully opened cotyledons in wild type, *fae1*, two *pCam5* lines (1–16-7, 1–12-8), and two *pCam5-atfae1* lines (28–3, 5–10). The emergence of the radicle was counted as germination. The appearance of roots and green fully opened cotyledons was scored as healthy seedlings (or established seedlings). Data represent means of three biological replicates ± SEM

To test the effect of HFA on seed germination and seedling development, seed germination rate and percentage of healthy seedlings (seedlings with open cotyledons) are counted. The time to reach 50% of the maximum germination (T50) was observed in the following equal or ascend order: WT (36 h) = *fae1* (36 h) = *pCam5* 1–12-8 (36 h) = *pCam5-atfae1* 5–10 (36 h) < *pCam5-atfae1* 28–3 (48 h) < *pCam5* 1–16-7 (60 h) (Fig. 8a). The time to reach 50% of the maximum number of healthy seedlings is in the following ascend order: WT (60 h) < *fae1* (72 h) < *pCam5-atfae1* 5–10 (84 h) < *pCam5* 1–12-8 (96 h) = *pCam5-atfae1* 28–3 (96 h) = *pCam5* 1–16-7 (96 h) (Fig. 8b).

### Comparison of seedling growth and mucilage among WT, *fae1* and *pCam5-atfae1*

**Fig. 9:**
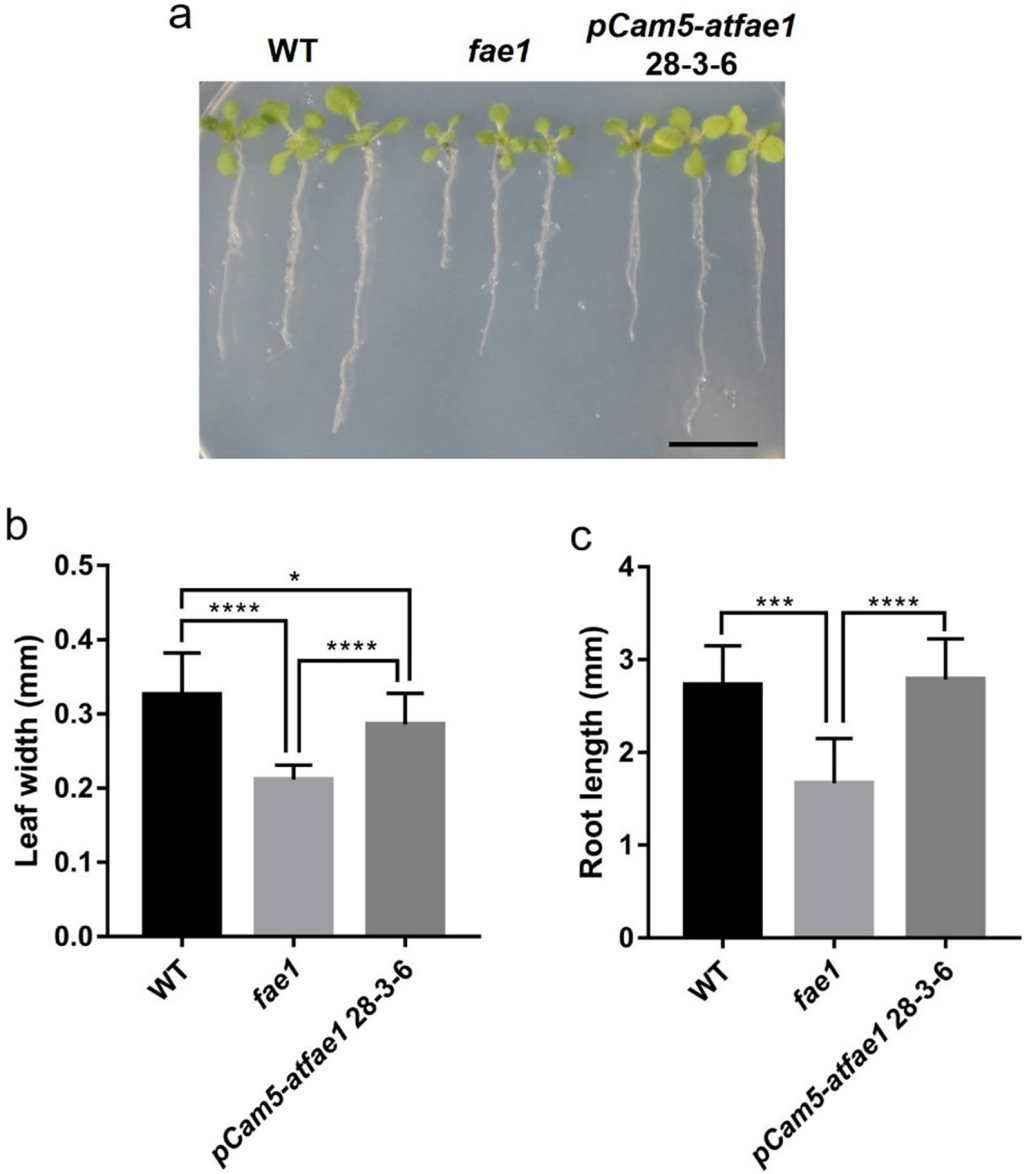
Seedling phenotype of *pCam5-atfae1* 28–3-6. **a** Seedling growth phenotype. The picture was taken for 10-day-old seedlings grown on ½ MS media (*n* = 3). Scale bars = 10 mm. The width of the true leave (**b**) and root length (**c**) were measured by Image J program (*n* = 3). Data represent means of three biological replicates ± SEM. Statistical analysis is one-way ANOVA with Turkey’s multiple comparison test (*p < 0.05, ***p < 0.001, ****p < 0.0001)

**Fig. 10:**
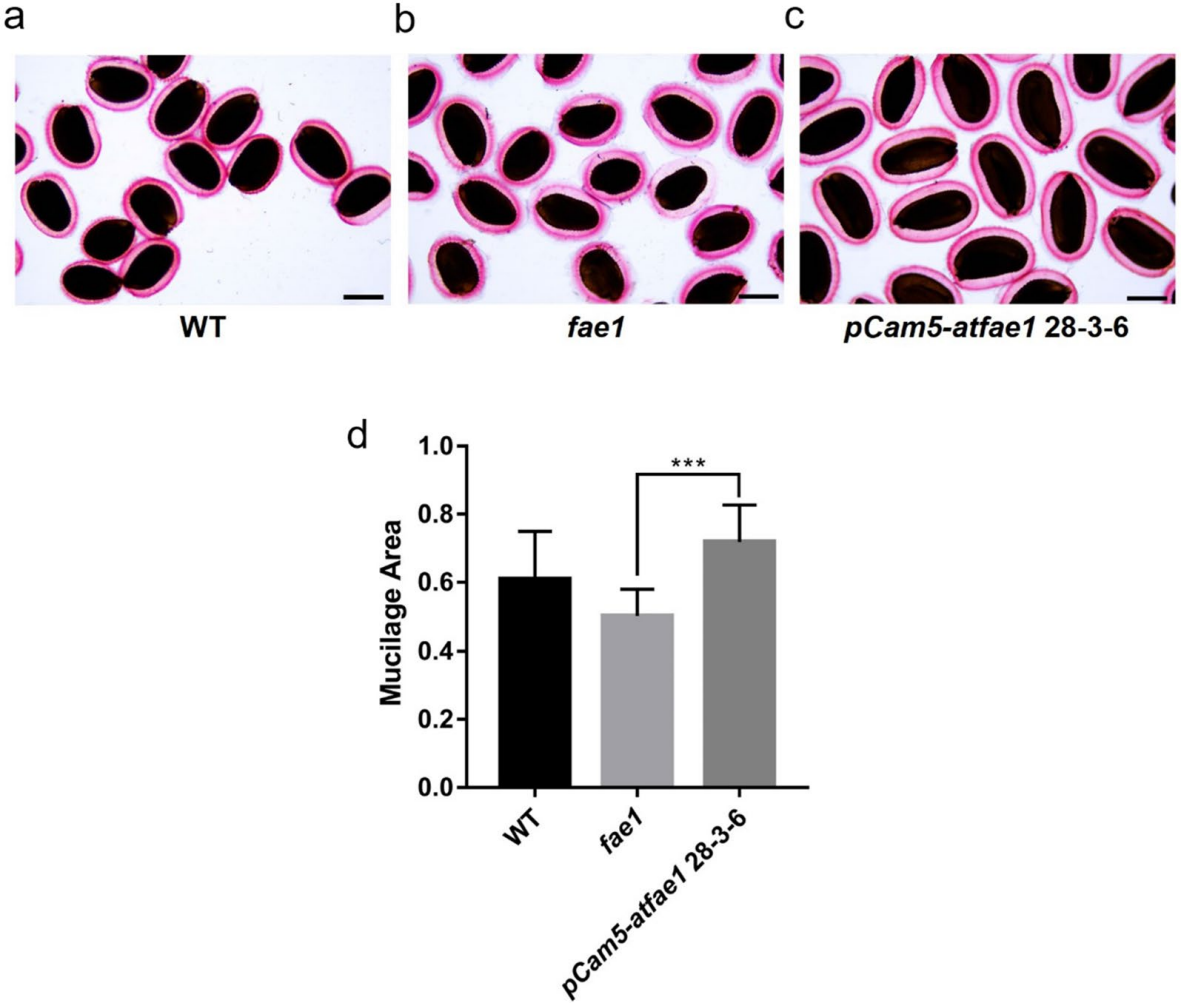
Analysis of mucilage by ruthenium red (RR) staining. Seeds of wild type (**a**), *fae1* (**b**)*,* and *pCam5-atfae1* 28–3-6 (**c**) were stained with RR (*n* = 50). Scale bars = 250 µm. **d** Mucilage area was calculated by HKBasic program using Nikon Eclipse Ci–L microscope (*n* = 10). The mucilage area was divided by the seed area for normalization (*n* = 10). Data represent means of three biological replicates ± SEM. Statistical analysis is one-way ANOVA with Turkey’s multiple comparison test (***p < 0.001)

To further examine the seedling growth, the leaf size and root length of the seedlings after 10 days of imbibement were measured for WT, *fae1* and *pCam5-atfae1*. As shown in Fig. 9, *fae1* had smaller leaf size and shorter root length than WT (Fig. 9). However, the leaf size of *pCam5-atfae1* is between WT and *fae1*, and the root length of *pCam5-atfae1* is statistically same as the of WT (Fig. 9). These results indicate that *pCam5-atfae1* restored the growth inhibition by *fae1* to a level close to WT. It is reported that transgenic *Arabidopsis* expressing a T-6b oncogene from *A. tumefaciens* increased seed size and oil content in mature seeds but decreased seed starch and seed coat mucilage content at the same time [54]. We compared mucilage content among WT, *fae1* and *pCam5-atfae1*. As shown in Fig. 10, the mucilage content in *fae1* was reduced compared to that of WT, but in *pCam5-atfae1*, the mucilage content was restored to WT level (Fig. 10). The results indicated that *pCam5-atfae1* increased size but at the same time also increased seed coat mucilage content.

## Discussion

The current commercial source of HFA is castor oil and its production is limited by undesirable agronomic traits [6]. To investigate if high levels of HFA can be produced in a common oilseed crop, we targeted multiple genes through different approaches. We devised a five-gene stacker, *pCam5*, that contained *RcFAH12, RcDGAT2, RcPDAT1-2, RcPDCT* and *RcLPCAT*. These genes encode key enzymes in the synthesis and incorporation of HFA into TAG (Fig. 1). We introduced *pCam5* into *Arabidopsis* which has been used as a model to study non-native HFA accumulation in seed oil. Homozygous transgenic *Arabidopsis* lines expressing *pCam5* produced total HFA at averages of 24–25.3% (Additional file 1: Table S3), which is comparable to previously reported HFA levels at 25–27% through co-expressing of single *RcDGAT2* [41], *RcPDAT1-2/RcPDAT1A* [21, 26, 27], or *RcPDCT* [21] in CL37. However, CL37 is generated in the *fae1* background, so we assessed the effect of the elongase by knocking out *AtFAE1* in *pCam5* transgenics through gene editing. The *pCam5-atfae1* lines increased total HFA content up to 29.1% (± 1.4% SD) (Additional file 1: Table S6; Fig. 5). Compared among CL37, *pCam5* and *pCam5-atfae1*, 20:1OH and 20:1 were found only in *pCam5* lines at averaging 3.1–3.4% and 12.7–13.4%, respectively (Additional file 1: Table S7; Fig. 5), indicating that the mutation of *fae1* in CL37 or deletion of *AtFAE1* in *pCam5-atfae1* prevented the elongation of 18:1OH to 20:1OH and 18:1 to 20:1, respectively. Considering similar levels of 18:2OH (averages 3.4–4.2%) displayed in *pCam5* and *pCam5-atfae1* (Additional file 1: Table S7), the increased total HFA in *pCam5-atfae1* was due to the increased 18:1OH content which rose from averages of 18.4–20.4% in pCam5 to averages 24.6–25.8% in *pCam5-atfae1* (Additional file 1: Table S7). The results indicated that efficient incorporation of 18:1OH–CoA into TAG occurred in *pCam5-atfae1* and the increased 18:1OH was at the expense of 20:1OH. For non-HFA, the blocked production of 20:1 in *pCam5-atfae1* coincided with the increases of 16:0 from 9.6–9.7% to 12.1–12.4%, 18:1 from 19.7–20.2% to 23.9–26.9%, and 18:2 from 17.1–18.5% to 18.5–21.2% (Additional file 1: Table S7). It is expected that knocking out of *AtFAE1* shifted 20:1 to 18:1. Since 18:1 was the substrate for FAD2 for synthesizing 18:2, an increase of 18:2 was also expected. However, the mechanisms of the increase of 16:0 were less clear. Based on 16:0 content in WT at 8.1% and in *fae1* at 11.1% (Additional file 1: Table S4), similar levels were observed in *pCam5* at 9.6–9.7% and in *pCam5-atfae1* at 12.1–12.4% (Additional file 1: Table S7). The increase in 16:0 would not be directly associated with the expression of castor genes, it could be due to the changes that occurred in plastid during FA synthesis and/or in exporting of 16:0 to cytosol and ER for TAG assembly. We observed that *pCam5-atfae1* lines dramatically increased seed size and weight. *pCam5-atfae1* 28–3-6 showed 1.8-fold bigger or heavier than CL37, 1.5-fold bigger and 1.8-fold heavier than *pCam5* 1–16-1–7, and 1.4-fold bigger or heavier than WT (Fig. 7). Total FAME per seed was measured 1.8-, 1.7- and 1.2-fold higher than that of CL37, *pCam5* 1–16-1–7 and WT, respectively (Fig. 6b). The dramatic increases in seed size and weight indicated that the five castor genes (*RcFAH12, RcDGAT2, RcPDAT1-2, RcLPCAT, RcPDCT*) over-expressed and *AtFAE1* silenced in *pCam5-atfae1* lines co-ordinately may enhance the seed development and reserve accumulation, surpassing WT.

Increases of de novo FA biosynthesis and input of FA flux from plastid to ER must occur to support seed growth of *pCam5-atfae1*. Overexpression of *RcFAH12* alone in WT* Arabidopsis* or various mutant backgrounds including CL37 accumulates limited HFA at approximately 17% in the seed oil and concomitantly reduces seed oil and weight [21, 26, 38–40, 42, 44] (Table 1). *Arabidopsis* utilizes mainly PC-derived DAG(2) to produce TAG [20, 55] (Fig. 1). The PC-derived DAG(2) is synthesized through de novo DAG → PC → PC-derived DAG(2) [28] (Fig. 1). When *RcFAH1*2 is expressed in *Arabidopsis* seeds, the de novo DAG containing 18:1OH is not efficiently converted to 18:1OH–PC by the *Arabidopsis* gene (e.g., *AtPDCT*), thus the converting step is a bottleneck to TAG synthesis. Over-expression of *RcPDCT* in CL37 increases HFAs from 17% to 25–27% and recovered seed oil content as well [21] (Table 1). Besides, the last step of TAG synthesis catalyzed by DGAT or PDAT is also bottlenecks in CL37, as overexpression of *RcDGAT2* or *RcPDAT1A* increases HFA content and recovers seed oil content [26, 27, 41]. *RcPDCT*, *RcDGAT2* and *RcPDAT1A* have been demonstrated to facilitate the utilization of 18:1OH to TAG through channeling 18:1OH–CoA, 18:1OH–DAG, and 18:1OH–PC to TAG [21, 26, 27, 41]. Metabolic analysis reveals that feedback inhibition of de novo FA synthesis, mainly through post-translational inhibition of acetyl-CoA carboxylase is triggered by unutilized HFA during TAG assembly, leads to reductions in HFA level and seed oil [46]. In fact, more efficient utilization of HFA for TAG synthesis by co-expression of *RcDGAT2* or *RcPDAT1A* in CL37 alleviates the reduced acetyl-CoA carboxylase (ACCase) activity [46]. Compared with *pCam5*, *pCam5-atfae1* accumulated more 18:1OH which is favorable substrate to castor genes. It is plausible that in *pCam5-atfae1* lines, the multiple bottlenecks were simultaneously overcome because of more efficient channeling of 18:1OH into TAG by *RcPDCT, RcDGAT2,* and *RcPDAT1-2*, which enhanced de novo FA synthesis and led to the increased TAG assembly and seed development.

Genetic modifications in *pCam5-atfae1* may provide additional metabolic enzyme complexes (metabolons) favorable in channeling C_18_ FA into TAG. In WT *Arabidopsis*, AtDGAT1 is the major enzyme that can rapidly utilize the PC-derived DAG(2) for TAG synthesis [28] (Fig. 1). Based on protein–protein interactions, AtDGAT1 interacts with AtPDCT and AtLPCAT2 [28], which forms a metabolon. The forward reaction of LPCAT transfers the acyl chain from CoA to LPC [15, 56], and the reverse LPCAT reaction can directly produce acyl-CoA [18] to be utilized for TAG biosynthesis (Fig. 1). PDCT carries out head group exchange between PC and DAG and is the key enzyme responsible to generated PC-derived DAG(2). In *pCam5-atfae1*, RcLPCAT and RcPDCT could also interact with AtDGAT1 forming additional metabolons. Besides, the absence of AtFAE1 would eliminate the competition with LPCATs for substrate C_18_ FA–CoA, allowing more C_18_ FA to be transferred to PC by LPCATs. Furthermore, RcLPCAT or RcPDCT has been shown strong activity in utilizing C_18_ FA [18, 22]. These features may collectively enable AtDGAT1 to assemble more PC-derived DAG(2) into TAG, resulting in increased seed oil biosynthesis. The PC-derived DAG(2) can turn to a larger and more slowly turned over bulk PC-derived DAG(3) pool, which is kept in equilibrium with PC by PDCT [28]. In the null mutant *dgat1-1*, AtPDAT1 becomes a major enzyme transferring FA from PC to form PC-derived DAG(3) [28] for TAG assembly [17, 57]. When *RcDGAT2* is overexpressed in *dgat1-1*, RcDGAT2 competes with AtPDAT1 for PC-derived DAG(3) [28]. Since RcDGAT2 interacts with AtPDCT, AtPDAT1, and AtLPCAT2 [28], it was likely additional metabolons were formed in *pCam5-AtFAE1* by interactions among enzymes from castor (RcDGAT2, RcPDAT1-2, RcPDCT, RcLPCAT) and *Arabidopsis* (AtPDAT1, AtPDCT, AtLPCAT2). These metabolons may efficiently utilize PC-derived DAG(3) for enhanced TAG accumulation in *pCam5-atfae1*. Although the exact mechanisms need to be investigated, *pCam5* and *pCam5-atfae1* provide important materials for metabolic studies to understand the metabolic complexes, substrate preference and pools, and pathways for TAG synthesis in seed oils.

We compared seed germination and seedling establishment among WT, *fae1*, *pCam5*, and *pCam5-atfae1* (Fig. 8). Although seed germination and seedling establishment of *pCam5* and *pCam5-atfae1* lines were slower than that of WT and *fae1*, they all had a 92–100% germination rate in 4 days (Fig. 8), a reasonable time required for *Arabidopsis* seed germination [58]. After 6 days of seed imbibition, seedling establishment in *pCam5* and *pCam5-atfae1* reached 77–83% and 81–91%, respectively, slightly lower than that of WT or *fae1* at 97% (Fig. 8). The results indicate that seeds from *pCam5* and *pCam5-atfae1* are viable. We also compared seedling growth (Fig. 9) and seed coat mucilage content (Fig. 10) among WT, *fae1* and *pCam5-atfae1*. Our results indicated that *pCam5-atfae1* recovered the adverse effect of *fae1* on seedling growth and seed coat mucilage content. Therefore, *pCam5-atfae1* increased seed size and oil content but displayed normal seedling growth and mucilage content. We are currently investigating if other carbohydrates such as sugar or starch are also proportionally increased in *pCam5-atfae1 *seeds.

A *pCam5-atfae1* line increased seed biomass and seed size by 40% compared to WT (Fig. 7). Before this research, the highest amount of HFA per seed is 1.81 µg HFA/seed [41] (Table 1), a *pCam5-atfae1* line produced 1.99 µg HFA/seed, the highest amount of HFA in a seed ever achieved. Ectopic expression of an *Agrobacterium tumefaciens* T-DNA oncogene, *T-6b*, also increases *Arabidopsis* seed biomass, oil content and seed size by 10% [54]. This is due to, in part, the expression of *T-6b* resulting in increased expression of *WRI1* and *DGAT1* and ultimately increasing the seed TAG [54]. Until now, only Camelina has been transformed with castor *FAH12*, the resulted plants produce 15% HFA [59]. By co-expression of *RcFAH12* and a lesquerella *KCS18* (or *PfKCS3* and *PfFAE1*) [52], the resulted transgenics produce up to about 20.9% of HFA [60]. The similarity between *Arabidopsis* and Camelina as hosts to express castor gene for HFA production would allow to apply knowledge from *Arabidopsis* to other crops, including Camelina, rapeseed, and soybeans. Besides, the design of *pCam5* allows one transformation process to express five different key genes essential for HFA synthesis, which avoids two or more separated procedures and thus shorten the time to generate HFA-producing plants. Our strategies demonstrated in this study can help design future HFA-producing crops through genetic engineering.

## Methods

### Plant materials, *Arabidopsis* transformation and growth

*Arabidopsis thaliana* wild type, Columbia-0 (Col-0), was used for the transformation of recombinant vectors. CL37 transgenic plant producing 17% HFA was used as the control [38]. Wild type and transgenic plants were germinated in MS medium containing 1% sucrose, the seedlings were transferred to soil or were directly germinated, and then grown in soil. *Arabidopsis* was grown on a control growth chamber under the conditions of 22 °C, 16 h light/8 h dark photoperiod. The light intensity was 100 µmol m^–2^ s^–1^. *Agrobacterium* strain, GV3101, was used to transform *pCam5* (pCambia-set1-set2-set3-set4-set5) vector into *Arabidopsis* Col-0 by floral dipping. The harvested seeds were selected on MS medium supplemented with 1% (w/v) sucrose, 10 mg/L DL–phosphinothricin (PPT), 100 mg/L of carbenicillin and 0.8% (w/v) plant agar. Selected T1 individuals showing resistance to PPT were transferred to soil for further experiments. T2 seeds and their decedents T3 and T4 were continued for PPT selection. As T2 seeds are segregating population, homozygous lines were identified by selecting T3 seeds with resistant to PPT and higher concentration of HFA than their segregants. Stable homozygous lines were further confirmed at T4 generation.

### Plant expression vector construction for co-expression of five genes

A vector containing five castor genes was constructed as the following method. Five castor genes involved in HFA synthesis and accumulated in TAG were contained in a vector; each gene has each promoter and terminator. Each of the cDNA *RcFAH12*, *RcPDAT1-2*, *RcDGAT2, RcPDCT* and *RcLPCAT* were synthesized from mRNA isolated from castor developing seeds and cloned into pENTR/D-TOPO vector (Invitrogen, USA) to obtain pENTR–RcFAH12, pENTR–RcPDAT1-2, pENTR–RcDGAT2, pENTR–RcLPCAT and pENTR–RcPDCT, respectively. To minimize the gene silencing, three kinds of seed-specific promoters including phaseolin promoter from soybean, *Arabidopsis* FAE1 promoter from *Arabidopsis* and Brassica Napin promoter from Brassica napus were used [61–63]. These promoters were amplified by PCR with the promoter cloned using vectors pGEM–phaseolin, pMDC32–AtFAE1, and pMDC–Napin as templates, respectively. Nos and Pin II were used as two types of terminators. These sequences were obtained by PCR amplification of the PinII terminator from the pBI221 vector and Nos terminator from the pPZP–3'PINII-Bar vector. Primers were designed to ligate the promoter, gene, and terminator at once (Additional file 1: Table S8). Each of the PCR products was inserted into the pUC19 vector by the infusion cloning method. Five subcloning vectors were completed after sequencing. Each gene expression set (promoter–gene–terminator) in five subcloning vectors was PCR-amplified using primers with unique restriction enzymes and cloned into the restriction site of the multi cloning site of the pCambia3300 plant expression vector stepwise. First, the Phas-FAH12-Nos set was inserted into *Eco*RI site of pCambia3300 and cloned to complete pCambia3300–Set1. Second, pCambia3300–set1–set2 was constructed by inserting the AtFAE1–RcPDAT1-2–PinII set into the *Sma*I site of pCambia3300–set1. Next, the Napin–RcLPCAT–Nos set was inserted into *Xba*I site to construct pCambia3300–set1–set2–set3. In addition, the pCambia3300–set4 was prepared by inserting the Phase-RcDGAT2–PinII set into the *Eco*RI*/Bam*HI site in pCambia3300, and the AtFAE1–RcPDCT–Nos set was further inserted into the *Bam*HI*/Hin*dIII site to prepare pCambia3300–set4–set5. To insert 5 gene expression sets into one vector, we cut the *Apa*I*/Hin*dIII in pCambia3300–set4 with *Apa*I*/Eco*RI in the vector of pCambia3300–set1–set2–set3 using *Apa*I site on one side and blunt-end ligation to construct pCambia3300–set2–set3–set4. In this vector, Phas-FAH12–Nos set1 was inserted into the *Eco*RI site again to construct pCambia2200–set1–set4–set2–set3. Finally, we constructed the pCambia3300–set1–set4–set2–set3–set5 (*pCam5*) by inserting the AtFAE1–RcPDCT–Nos set into the *Asc*I site of pCambia3300–set1–set4–set2–set3.

### CRISPR/Cas9 vector cloning for *ArabidopsisFAE1* knockout

To knockout the *Arabidopsis* *fatty acid elongase 1* (*FAE1)* gene, two guide RNAs (5′-GCTGCAAAAGTCTTCCGCGG-3′ and 5′-AAGTTAACCCTAGAGAGATC-3′) were designed using the CRISPR–RGEN tool available online (http://www.rgenome.net). The *Bsa*I enzyme site was attached to the front of each primer and PCR was performed using the pCBC–DT1T2 vector as a template. The PCR product was purified by a PCR Purification kit (QIAGEN, Germany). This product was put into pHEE401E-egg vector and treated with *Bsa*I enzyme to proceed with the golden gate reaction [53]. After cloning, Sanger sequencing was performed to confirm whether the guide RNA sequence was in the vector. This vector was transformed into *pCam5* lines producing 25% HFA in seed oil.

### Fatty acid analysis

The fatty acid composition and total oil content of *Arabidopsis* seeds was analyzed using gas chromatography (GC). First, 500 μl toluene and 500 μl of 5% H_2_SO_4_ dissolved in methanol were added and methylation was performed in 85 °C water bath. Pentadecanoic acid (15:0) was used as the internal standard. After methylation at 85 °C for 2 h, the sample was cooled and 1 ml of 0.9% NaCl was added. Then 1 ml of *n*-hexane was added and violently mixed. The sample tube was centrifuged at 3000 rpm for 2 min. The supernatant was transferred to a new 6ml round tube. The step from adding 1 ml of n-hexane to transferring supernatant was repeated three times. After total of 3 ml supernatant was purged with nitrogen gas, the 200 μl of n-hexane was added thereto. The FAMEs dissolved were analyzed by GC-2010 plus (Shimadzu, Japan) with flame ionization detector and DB-23 column (30 m × 0.25 mm, 0.25 $\mathrm{\mu m}$ film, Agilent, USA). The range of oven temperature is from 190 °C to 230 °C at 3 °C/min.

### Measure of seed length, width, size, and weight

*Arabidopsis* seed weight was measured using an electronic scale (OHAUS, USA) capable of measuring up to the fourth decimal point. The seed weight (*n* = 100) was measured, followed by 3 repetitions, and then the mean values of the three lines were measured. To check the seed size, the length and width of 30 seeds were measured using an optical microscope (NIKON, Japan) in triplicate. Seed sizes were calculated by multiplying the width by length using the software, Image J (http://imagej.nih.gov/ij/).

### Measure of seed germination rate, cotyledon opening, seedling growth and mucilage

To measure the germination rate and seedling growth, the seed was sterilized in 70% ethanol for 1 min and 0.5% (w/v) NaOCl for 5 min and then washed in distilled water three times. The seeds were subjected to stratification at 4 °C for 3 days. Next, three repetitions of 30 seeds for germination test or 100 seeds for seedling growth observation were made on half strength of MS media containing 1% (w/v) sucrose and 0.8% (w/v) plant agar, and cultured in a culture chamber of 23 °C at 16 h light/8 h dark condition. The germination rate was measured at intervals of 12 h and confirmed up to 144 h. Germination rates were counted when a radicle emerged from the seed. Seedlings with two fully opened cotyledons at a 180-degree angle were considered healthy seedlings. The seedling growth was checked by measuring the leaf width in triplicate using the Image J program (*n* = 3). The three repetitions of 50 seeds were stained with 0.01% ruthenium red to check the mucilage of the seed coat. All experiment steps for mucilage analysis were followed by a previously published paper [64]. To quantify the mucilage area, mucilage area was calculated except for seed area by HKBasic program using Eclipse Ci-L microscope (NIKON, Japan) and divided by seed area (*n* = 10).

### Reverse transcriptase PCR and Quantitative RT-PCR

Total RNA was isolated from the developing siliques having different stages from 1 to 18 days after flowering. RNA extraction has followed the method mentioned in Onate-Sanchez et al. [65]. RNA concentration and quality were determined by measuring the A260/A280 ratio using a DS-11 spectrophotometer (Denovix, USA). RNA samples were treated with DNase I (Thermo Fisher Scientific, USA). 1 μg of cDNA was synthesized using a PrimeScript 1st strand cDNA synthesis kit (Takara, Japan) following the recommendation of the manufacturer. Total RNA was extracted in triplicate and RT–qPCR was performed in triplicate on each sample in StepOnePlus Real-Time PCR System (Thermo Fisher Scientific, USA) using SYBR green master mix (TOYOBO, Japan). The expression level was determined using the ΔΔCт method. The ΔCт value was obtained by subtracting the Cт value of the target gene and the Cт value of the endogenous control. In addition, we obtained ΔCт by subtracting the Cт value of the treated group and the Cт value of the control group. The relative expression levels were obtained by subtracting the two ΔCт values, which was a ΔΔCт. After that, the value of ${2}^{(-\Delta \Delta {\mathrm{C}}_{\mathrm{T}})}$ was obtained for a comparison of relative expression. eIF4a was used as a control gene to normalize relative expression level. For the expression of quantitative RT–PCR, the expression of each gene of DAF1 was set to 1. The primers of RT–PCR and RT–qPCR are described in Additional file 1: Table S9. The stages were divided into 6 stages for 18 days after flowering, indicating the seed development stage. For example, stage 1 indicates 1–3 days after flowering.

## Supplementary Information

**Additional file 1: Table S1.** Fatty acid composition of T2 seeds from *pCam5* T1 plants. **Table S2.** Fatty acid composition of T3 seeds from *pCam5* 1 and *pCam5* 4 independent T2 plants (n = T3 line number). **Table S3.** Fatty acid composition of T4 seeds from *pCam5* 1–12, *pCam5* 1–16 and *pCam5* 4–5 independent T3 plants (n = T4 line number)**. Table S4.** Fatty acid composition of T5 seeds from *pCam5* 1–12-8, *pCam5* 1–16-7, *pCam5* 1–16-8 and *pCam5* 4–5-2 independent T4 plants (n = T5 line number). **Table S5.** Fatty acid composition of T2 seeds *pCam5-atfae1* 1–12 and *pCam5-atfae1* 1–16 independent T3 plants (n = T2 *pCam5-atfae1* line number). **Table S6.** Fatty acid composition of T3 seeds from *pCam5-atfae1* 5, *pCam5-atfae1* 9 and *pCam5-atfae1* 28 independent T2 plants (n = T3 *pCam5-atfae1* line number). **Table S7.** Fatty acid composition of T4 seeds from *pCam5-atfae1* 5–9, *pCam5-atfae1* 28–3 independent T3 plants (n = T4 *pCam5-atfae1* line number). **Table S8.** Promoter–gene–terminator was linked by infusion method and primer list used for pUC19 subcloning. **Table S9**. Primers were used in this study.

**Additional file 2: Fig. S1.** Expression pattern of five transgenes (*RcFAH12, RcPDAT1-2, RcPDCT, RcLPCAT and RcDGAT2*) in the developing seed in *pCam5* 1–16-8 (a, b) and *pCam5-atfae1* 5–9 line (c, d). *AtACT2* and *eIF4a* were used as controls for RT–PCR and RT–qPCR, respectively. *RcFAH12, RcPDAT1-2, RcPDCT, RcLPCAT* and *RcDGAT2* expression analyses were performed using their specific primers (Table S9). The stages were divided into 6 stages for 18 days after flowering (DAF). Stage 1: 1 ~ 3 DAF, stage 2: 4 ~ 6DAF, stage 3: 7 ~ 9 DAF, stage 4: 10 ~ 12 DAF, stage 5: 13 ~ 15 DAF, stage 6: 16 ~ 18 DAF.

## Data Availability

All data generated or analyzed during this study are included in this published article and its Additional files.

## Abbreviations

TAG: Triacylglycerol
acyl-CoA: Acyl-coenzyme A
FA: Fatty acid
HFA: Hydroxy fatty acid
DAG: Diacylglycerol
PC: Phosphatidylcholine
FAH12: Oleate $\Delta$ 12-hydroxylase
GPAT: Glycerol-3-phosphate acyltransferase
LPAT: Lysophosphatidic acid acyltransferase
DGAT: 1,2-*sn*-Diacylglycerol acyltransferase
PDAT: Phospholipid:DAG acyltransferase
LPCAT: Lyso-PC acyltransferase
PDCT: PC:DAG cholinephosphotransferase
FAE1: Fatty acid elongase 1
FAME: Fatty acid methyl ester
WT: Wild type
DW: Dry weight
ER: Endoplasmic reticulum
RT–PCR: Reverse transcription PCR
RT–qPCR: Quantitative reverse transcription PCR
