# Supplementary material for: Enhanced production of hydroxy fatty acids in Arabidopsis seed through modification of multiple gene expression
Source: Biotechnol Biofuels Bioprod. 2022 Jun 18;15:66. doi: 10.1186/s13068-022-02167-1 (PMC9206371; doi:10.1186/s13068-022-02167-1)
Supplement: Supplementary file 2 — Additional file 2: Fig. S1. Expression pattern of five transgenes (RcFAH12, RcPDAT1-2, RcPDCT, RcLPCAT and RcDGAT2) in the developing seed in pCam5 1–16-8 (a, b) and pCam5-atfae1 5–9 line (c, d). AtACT2 and eIF4a were used as controls for RT–PCR and RT–qPCR, respectively. RcFAH12, RcPDAT1-2, RcPDCT, RcLPCAT and RcDGAT2 expression analyses were performed using their specific primers (Table S9). The stages were divided into 6 stages for 18 days after flowering (DAF). Stage 1: 1 ~ 3 DAF, stage 2: 4 ~ 6DAF, stage 3: 7 ~ 9 DAF, stage 4: 10 ~ 12 DAF, stage 5: 13 ~ 15 DAF, stage 6: 16 ~ 18 DAF. [file 13068_2022_2167_MOESM2_ESM.pdf]

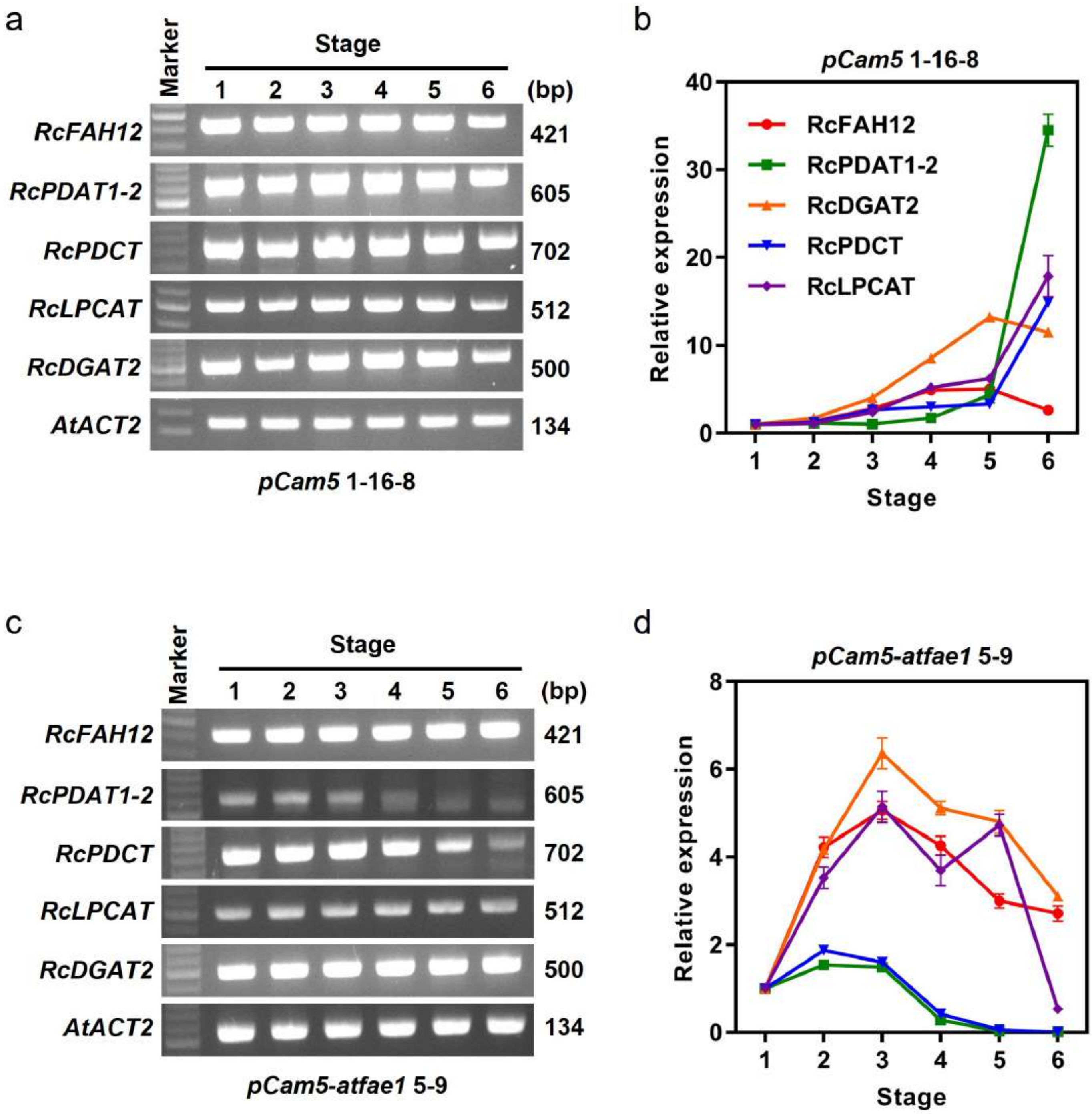

Fig S1. Expression pattern of five transgenes (*RcFAH12*, *RcPDAT1-2*, *RcPDCT*, *RcLPCAT* and *RcDGAT2)* in the developing seed in *pCam5* 1-16-8 (a-b) and *pCam5-atfae1* 5-9 line (c-d). *AtACT2* and *eIF4a* were used as controls for RT-PCR and RT-qPCR, respectively. *RcFAH12, RcPDAT1-2, RcPDCT, RcLPCAT* and *RcDGAT2* expression analyses were performed using their specific primers (Table S9). The stages were divided into 6 stages for 18 days after flowering (DAF). Stage 1: 1~3 DAF, stage 2: 4~6DAF, stage 3: 7~9 DAF, stage 4: 10~12 DAF, stage 5: 13~15 DAF, stage 6: 16~18 DAF
